# Exploring Splice-Site Mutations in LAMA2-Related Muscular Dystrophies: A Comprehensive Analysis of Genotypic and Phenotypic Patterns

**DOI:** 10.7759/cureus.61599

**Published:** 2024-06-03

**Authors:** Samira Nmer, Amina Ameli, Said Trhanint, Sana Chaouki, Laila Bouguenouch, Karim Ouldim

**Affiliations:** 1 Biomedical and Translational Research Laboratory, Faculty of Medicine, Pharmacy and Dentistry, Sidi Mohamed Ben Abdellah University, Fez, MAR; 2 Medical Genetics and Oncogenetics Unit, Central Laboratory of Medical Analyses, Hassan II University Hospital, Fez, MAR; 3 Pediatric Emergency, Hassan II University Hospital, Fez, MAR

**Keywords:** lgmd-like phenotype, mdc1a, whole exome sequencing, splice-site mutations, laminin-α2, lama2-related muscular dystrophies

## Abstract

LAMA2-related muscular dystrophies (LAMA2-RDs) constitute the most prevalent subtype of congenital muscular dystrophies (CMDs). The clinical spectrum of LAMA2-RDs exhibits considerable diversity, particularly in motor development and disease progression. Phenotypic variability ranges from severe, early-onset presentation, known as merosin-deficient CMD type 1A, to milder, late-onset presentations, including limb-girdle muscular dystrophy-like phenotype. In this study, whole exome sequencing (WES) was applied to a family with a single proband affected by severe muscular dystrophy. The identified causative mutation was a biallelic splice-site mutation in intron 58 of the *LAMA2* gene, leading to a premature termination codon in the critical G domain of laminin-α2 and resulting in a severe phenotype. Additionally, we summarized previously reported splice-site mutations to investigate the clinical and transcription consequences of these mutations. Our findings conclude that splice-site mutations predominantly lead to severe MDC1A, whether in a homozygous or heterozygous state, often associated with another loss-of-function mutation. Besides, splice-site mutations with available analysis of their transcriptional consequences were found to be responsible for exon skipping in most cases and the loss of the reading frame. These findings revealed the importance of WES in identifying disease-causing mutations, particularly in highly diversified pathologies like LAMA2-RDs. The results also underscore the importance of transcriptional analysis in determining the impact of splice-site mutations and the phenotype of LAMA2-RDs on patients.

## Introduction

Congenital muscular dystrophies (CMDs) are a group of neuromuscular diseases characterized by an early onset of muscular deficiency and dystrophic changes affecting muscles [1]. They comprise highly diversified subtypes of pathologies at the clinical and genetic levels [2].

LAMA2-related muscular dystrophies (LAMA2-RDs) represent the most frequently reported subtype of CMDs, explaining away 30% of the cases [3]. It is linked to mutations affecting the *LAMA2 *gene found at the locus 6q22-23, encoding the laminin alpha-2 chain (laminin-α2). In skeletal muscle fibers, laminin-α2 associates with β1 and γ1, the two light chains of laminin, forming a heterotrimeric isoform of laminin known as laminin 211 or merosin [2]. This extracellular protein, merosin, links the extracellular matrix to the two transmembrane proteins, α-dystroglycan and integrin α7β1, through laminin-α2. This linkage is crucial for adhesion, basement-membrane assembly, and downstream signaling events, ensuring muscle integrity and functioning [4].

Clinically, LAMA2-RDs are commonly categorized into two phenotypic groups: a severe, early-onset presentation with features resembling CMD, widely known as merosin-deficient congenital muscular dystrophy type 1A (MDC1A), and a milder, late-onset presentation displaying characteristics indicative of limb-girdle muscular dystrophy (LGMD) [5]. The classic severe LAMA2-RD phenotype is somewhat a homogenous CMD phenotype; it is mainly characterized by total laminin-α2 deficiency and typically manifests with hypotonia since birth and onset of symptoms within the first six months of life. Respiratory problems, as well as feeding difficulties, can also be present at birth. Developmental milestones are delayed, with the inability to attain independent ambulation. Other features include scoliosis, joint contractures, elevated creatine kinase (CK) levels (>1,000 IU/L), and typical white matter changes (WMC) on magnetic resonance imaging (MRI) [5-7]. Variability in this early onset LAMA2-RD is noteworthy since ambulation is attained in up to 10% of patients with complete laminin-α2 deficiency [8]. Moreover, partial expression of laminin-α2 is present in certain patients who may present consequently with a relatively milder phenotype [5]. On the other hand, mild LAMA2-RD is characterized by a partial expression of laminin-α2, delayed milestones, and a gain of ambulation [6]. It is, however, characterized by phenotypic variability, encompassing manifestations from CMD-like to milder and later presentations, including late-onset LGMD-like phenotype [5]. In this form of LAMA2-RD, scoliosis occurs less frequently than the severe form, but joint contractures are notable [5,6].

Genetically, as indicated in a review published in 2018, LAMA2-RDs are predominantly associated with single nucleotide variants (SNVs), followed by small deletions, small insertions, and, finally, large rearrangements such as deletions and duplications [9]. In the ClinVar database, more than 4000 unique variants have been identified in the *LAMA2 *gene with 168 splice-site pathogenic variants [10]. This makes splice-site mutations one of the frequently reported variants associated with LAMA2-RDs.

Throughout this paper, we aimed to report the diagnosis of a 20-month-old infant whose clinical and paraclinical examinations favored MDC1A. A splice-site mutation in the intron 58 of the *LAMA2 *gene was identified through whole exome sequencing. As splice-site altering mutation can induce different transcriptional outcomes that affect patients’ phenotype and disease severity, a review of the previously reported splice-site pathogenic variants was conducted.

## Case presentation

Patient, clinical data collection, and ethics

The patient underwent a comprehensive clinical examination and was assessed at the Medical Genetics and Oncogenetics unit at Hassan II University Hospital of Fez by a qualified clinician. The clinician thoroughly reviewed and documented the medical history, clinical data, and results of all paraclinical analyses that the patient underwent. Subsequently, written informed consent was obtained from the patient's parents. Finally, a peripheral blood sample was collected from the patient and the parents for genetic analysis.

Case description

The proband, a 20-month-old girl, is the offspring of second-degree consanguineous parents from the northeast of Morocco. According to her mother, the pregnancy was normal, although it was not regularly monitored, and the delivery was spontaneous with no complications. The patient displayed hypotonia since the neonatal period, followed by psychomotor retardation, which prompted the parents to seek medical consultation from a pediatrician at the age of eight months. She gained the ability to control her head at four months and could sit independently without support by the ninth month. This remained the latest developmental achievement observed at the most recent assessment.

The interrogatory revealed that she had three older siblings (one sister and two brothers) who died at the age of 13 years due to respiratory complications with a notion of psychomotor regression and loss of ambulation earlier in their lives. However, their diagnosis has never been established since the parents have never sought a medical examination.

Her clinical assessment indicated peripheral hypotonia without amyotrophy or calf hypertrophy. No facial weakness or ophthalmoparesis was observed. An equinovarus foot and scoliosis were noted. Additionally, she had neonatal pectus excavatum but no facial dysmorphism. A hemangioma was present on her neck. Further examinations confirmed normal cardiac and respiratory functions. Besides, as reported by the mother, she had never experienced seizures.

**Figure 1:**
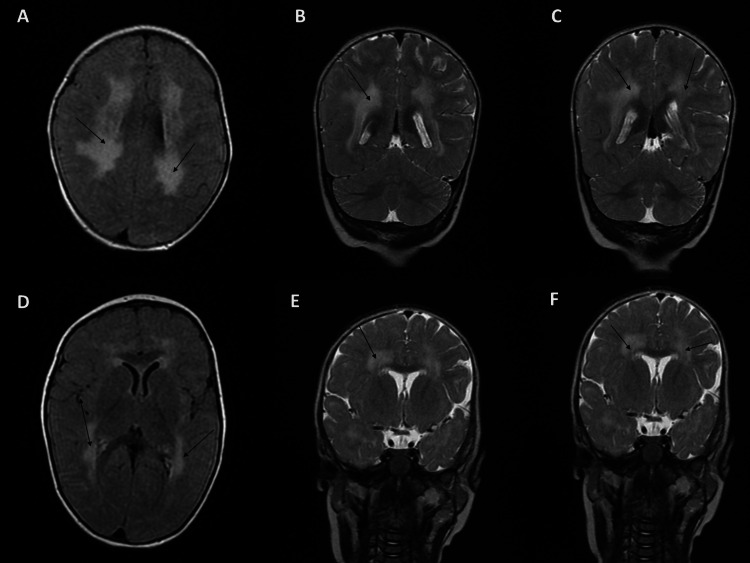
Results of the MRI scan of the case From A to F, the images show changes in the frontoparietal and periventricular white matter of the patient

Given the symptoms observed in the case, various analyses were undertaken to guide the diagnosis. At the age of eight months, CK analysis indicated a level of 1363 UI (9 times the normal range) (Table 1). The electromyogram revealed a myogenic pattern in the muscles of both the upper and lower limbs. Additionally, the MRI uncovered changes in the frontoparietal and periventricular white matter in the T2 MRI scan (Figure 1). Immunostaining and immunoblotting on muscle biopsy were not conducted due to the invasive nature of the procedure. Based on these findings, LAMA2-RDs were suspected, leading to the subsequent implementation of WES.

**Table 1:** CK analysis of the patient CK: Creatine kinase

	Result	Normal findings
CK	1363 UI/L	0.00-145.00

Genetic analysis

Genomic DNA extraction was conducted according to the manufacturer’s recommendations using the Invitrogen PureLink™ Genomic DNA Mini Kit (Thermo Fisher Scientific-USA). DNA quality and quantity were evaluated using a nanodrop spectrophotometer and a Qubit 3 Assay kit, respectively. The proband’s DNA was later subjected to WES conducted at the 3billion lab in Seoul, South Korea.

Following identifying the causative variant, Sanger sequencing was performed on the patient to verify the variant and on the parents for carrier screening, utilizing the BigDye™ Terminator v3.1 Cycle Sequencing kit (Applied Biosystems, Thermo Fisher Scientific).

Results

**Figure 2:**
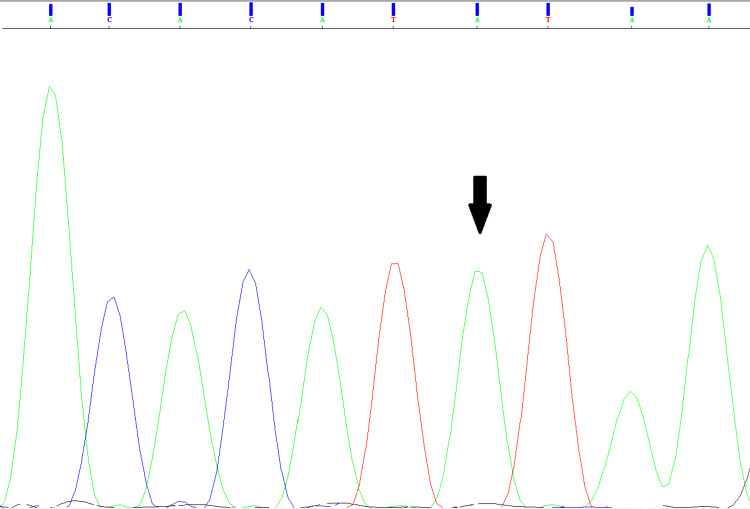
Sanger sequencing results of the case The electropherogram shows a single green peak at position 8244+1 (designated by the arrow) corresponding to a homozygous substitution of G with A.

**Figure 3:**
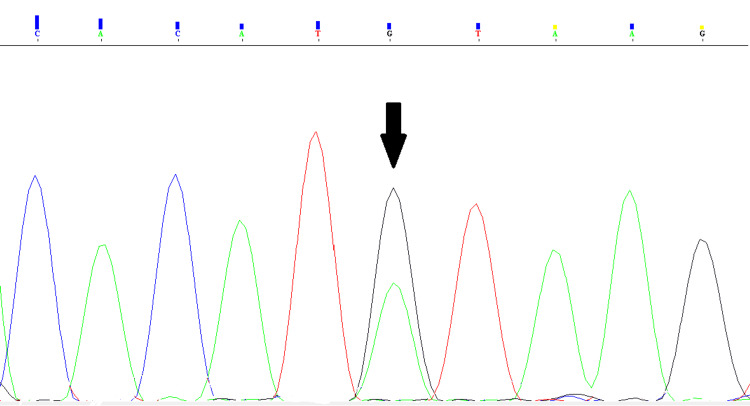
Sanger sequencing results of the parents of the case The electropherogram shows two peaks at position 8244+1 (designated by the arrow) corresponding to the nucleotides G and A, which demonstrates a heterozygous substitution of G with A.

Genetic analysis revealed that the causative pathogenic variant in the patient is c.8244+1G>A (NM_000426.4), a homozygous splice-site mutation located at the first position of the donor splice site in intron 58 of the *LAMA2 *gene (Figure 2). Sanger sequencing confirmed that the parents carried the mutation in a heterozygous state (Figure 3).

At the level of mRNA, this mutation, present at the highly conserved GU dinucleotide, affects the exon 58 splicing site selection and leads to its complete skipping; this skip disrupts the open reading frame, which eventually generates a premature termination codon (PTC) in exon 59 at position +34. At the level of protein- if translated, the PTC in exon 59 ends the translation with a truncated laminin α2 deprived of the C terminal amino acids residues, which cluster LG4 and LG5 domains: the functional binding region of laminin 211 to α-dystroglycan [11].

The c.8244+1G>A mutation has multiple entries in the LOVD database, which classiﬁed it as a pathogenic splice-site variant [12]. It is present in the normal population with a very low frequency (rs749522728, gnomAD, and ExAC 0.0008%) [13].

## Discussion

The present study reports the genetic analysis of a 20-month-old infant whose clinical profile, MRI images, and CK analysis were in favor of LAMA2-RDs. Because of the important similarities between CMDs and the molecular weight of the *LAMA2 *gene, the patient underwent WES, which confirmed the suspicion of LAMA2-RDs and was consistent with the clinical and paraclinical results. The incriminated pathogenic variant was c.8244+1G>A, located at the canonical donor splice site of intron 58. As mentioned, this mutation has already been documented in previous studies with multiple entries in the LOVD database that classified it as pathogenic [12].

Given the severe phenotype and early onset symptoms observed in our patient, it was deduced that she had a severe MDC1A. Her inability to achieve independent sitting aligns with the literature's findings, as most MDC1A patients face challenges in attaining independent sitting, and fewer than 10% can walk without support [8,14].

The case in our study presented with WMC, aligning with current knowledge, as the majority of patients above six months of age typically display typical WMC on MRI, manifested by abnormal T2 signals in the periventricular and subcortical white matter [7,15]. These changes are linked to increased brain water content, which is, in turn, due to leaky basal laminar connections of the blood-brain barrier (BBB). In essence, laminins are part of a vital “outer barrier” of mature BBB: the gliovascular basal lamina. The interaction of laminin with dystroglycan at the gliovascular interface is paramount as it regulates BBB's structure, maturation, and function, which discerningly regulates central nervous system (CNS) homeostasis. MRI irregularities found in LAMA2-RDs are at least in part due to BBB disturbance [16].

Furthermore, this infant had never experienced any episode of seizures, nor did she exhibit intellectual disability. Intellectual disability is another symptom documented in a few cases [6,7,17], though most patients generally showed normal cognition. Moreover, seizures, with or without structural brain lesions, were reported in up to 17% of MDC1A cases [6,7,17].

The reported mutation was first discovered in a Tunisian patient with a homozygous genotype and a severe MDC1A phenotype. The case in that study benefited from muscle biopsy, which revealed a complete deficiency in merosin protein; no laminin-α2 immunostaining was observed [18]. Though our patient did not undergo an analysis of the level of expression of merosin, patients with MDC1A are known to have a total absence of the protein, aside from some exceptions [5,6,17]. This could be explained by two different mechanisms: degradation of the nonsense mRNA through the nonsense-mediated mRNA decay mechanism [11,19] or the degradation of the aberrant laminin α2 after its generation by the so-called “protein quantity control” mechanism [11,20].

Four individuals among the previously documented patients with this mutation, and with available clinical data, exhibited a severe phenotype regardless of whether the mutation was present in the homozygous or compound heterozygous state. However, the two patients with a homozygous genotype showed complete deficiency in laminin-α2, whereas the other two cases with compound heterozygous mutations exhibited a partial expression of it [6,11,18]. The impact of the second mutation could explain this difference in the expression of laminin-α2. Indeed, all the patients reported with c.8244+1G>A mutation had a severe MDC1A phenotype. Moreover, other pathogenic splice-site mutations affecting the same position (c.8244+1) were documented to be linked to the MDC1A phenotype [9]. This suggests that this position is a crucial functional splicing site within the *LAMA2 *gene. 

Hitherto, 168 *LAMA2 *splice-site variants, clinically classified as pathogenic or likely pathogenic, are listed in the ClinVar database [10], indicating thus that splice-site mutations serve as a crucial mechanism contributing to LAMA2-RDs. A splice variant modifies the DNA sequence at the splice site, delineating the boundary between exons and introns. This modification can result in the inclusion of introns, the loss of exons, and a change in the protein-coding sequence, thereby disrupting the process of RNA splicing [21]. It can disrupt or diminish mRNA processing via exon skipping or cryptic splice-site activation [22].

In the *LAMA2* gene, splice-site mutations are distributed throughout the entire gene without being linked to any specific mutational hotspot. These mutations are predominantly present in introns' first and last two conserved nucleotides. Following a comprehensive review of all the published mutations, we identified 57 pathogenic variants with available clinical data of the reported individuals. These variants are detailed in Table 3 in Appendices.

The 57 mutations were identified in 97 patients. Regarding genotypes, 24 patients displayed homozygous mutations, while 73 cases exhibited compound heterozygous mutations. Additionally, in four cases, only one heterozygous mutation was identified. Besides, four mutations were identified in many patients: c.3924+2T>C (n=11), c.5234+1G>A (n=11), c.5562+5G>C (n=7), and c.8244+1G>A (n=11). This observation may be ascribed to the high recurrence of these mutations widely or among patients from particular populations. The c.3924+2T>C mutation has been exclusively identified in patients from the Middle East or Sudan, indicating a likely founder effect in this population [23,24]. Therefore, the suspicion of LAMA2-RDs in patients of this origin should prompt a search for this specific mutation. The c.5234+1G>A mutation was identified in five patients from Portugal and seven from Brazil [25-27]. The c.5562+5G>C variant was predominantly observed in patients from the UK [6,28,29]. Lastly, the mutation c.8244+1G>A (n=9) was detected in five Brazilian patients, two from Tunisia, two from Morocco, and two others with unspecified origins [6,11,18,26,30]. This suggests the possibility of it being a recurrent mutation, particularly in the Maghreb region.

Regarding the clinical presentation, patients were divided into two main categories: MDC1A cases, characterized by symptoms appearing within the first year of life (such as hypotonia, delayed motor development, or both), and LGMD-like phenotype cases, where symptoms began beyond the first year, with normal motor milestones and independent ambulation [17]. Further classification within the MDC1A group included severe MDC1A phenotype, where ambulation was never achieved or achieved but lost within the first decade of life, and mild MDC1A phenotype, where ambulation was attained and maintained beyond the first decade until the last observation. Thus, 87 patients were categorized as MDC1A patients. From them, 68 patients were classified as severe MDC1A cases, and 19 individuals were considered mild MDC1A cases. Besides, 10 patients were identified as LGMD-like phenotype cases. Among individuals with severe MDC1A (n=68), 16 patients had a homozygous genotype, 50 patients exhibited a compound heterozygous genotype characterized by a splice-site mutation combined with another loss-of-function mutation (splicing/frameshift/nonsense), and only two patients had a splice-site mutation associated with a missense mutation [6,7,11,17,18,24-26,30-41]. Of the 68 severe MDC1A cases, 32 patients had documented results regarding the level of laminin-α2 expression. Within this group of 32 patients (32/68), 23 individuals exhibited complete deficiency, while only 9 demonstrated partial expression. On the other hand, all patients with mild MDC1A (n=19) exhibited a genotype comprising either homozygous splice-site mutations or a combination of a splice-site mutation with either a nonsense mutation, a frameshift mutation or another splice-site mutation [6,17,23,24,26,28,29,42,43]. Within the group of mild MDC1A, data on laminin-α2 expression were available for ten patients (10/19): 9 patients exhibited a partial expression of laminin-α2, while only one patient showed complete deficiency. This suggests that genotypes characterized by homozygous splice-site mutations or biallelic loss-of-function mutations (splicing with splicing/frameshift/nonsense) are primarily associated with the manifestation of severe early-onset MDC1A, especially when combined with a complete absence of laminin-α2. Conversely, a milder form of early-onset MDC1A is predominantly observed when these genotypes are linked to residual expression of laminin-α2.

Concerning the patients with LGMD-like phenotype (n=10), their genotype was primarily made of splice-site mutations with missense mutations (7/10) [17,26,27,44-47]. Genotypes with missense mutations, especially those in the N-terminal region with a preserved C-terminal expression (G domain of laminin-α2), are linked to partial expression of laminin-α2 and less severe clinical manifestations [5,9], which could explain these LGMD-like phenotype cases. Additionally, an individual exhibiting the LGMD-like phenotype (1/10) harbored a splice-site mutation that resulted in the loss of the reading frame and an in-frame duplication [46]. This phenotype could be elucidated by the preservation of the reading frame due to the in-frame duplication (exon 4-12), potentially allowing for the translation of an abnormal yet partially functional laminin-α2. Unfortunately, laminin-α2 expression was not assessed to substantiate this hypothesis further. Besides, one of the LGMD-like phenotype cases (1/10) had a genotype made of a splice-site mutation and an out-of-frame deletion [46]. The pathogenicity of the reported splice-site mutation remains unclear, and there is a lack of information regarding the level of laminin-α2 expression to elucidate the phenotype.

Considering the affected domain in laminin-α2, 50% of patients with MDC1A phenotype (44/87) exhibited mutations in the G domain. In contrast, none of the patients with LGMD-like phenotype had mutations affecting this domain, suggesting thus that an aberrant G domain in laminin-α2 causes a severe phenotype. The G domain is indispensable for promoting the connection of Laminin 211 to muscular cells through surface receptors, specifically dystroglycan and integrin α7β1. This connection has been confirmed to be crucial for muscle integrity and the normal functioning of muscular cells [4]. The mutation c.8244+1G>A, which disrupted the normal splicing of mRNA and induced a premature termination codon (PTC) at this domain, was documented to cause a severe MDC1A with a total absence of laminin-α2 [11]. This underscores the importance of the G domain in laminin-α2, elucidating thus the severity of the phenotype observed in individuals deficient in it.

Genotypes consisting solely of splice-site mutations, either in the homozygous or compound heterozygous state, accounted only for MDC1A cases. These genotypes correlated with 41% of MDC1A cases (36 out of 87), which reaffirms the involvement of this specific type of mutation in MDC1A and emphasizes the importance of developing strategies for the functional analysis of these splice-site mutations. Such strategies would enhance the establishment of a genotype/phenotype correlation in LAMA2-RDs. Unfortunately, no study has been dedicated to the functional analysis of splice-site mutations in the *LAMA2 *gene. Furthermore, only a few studies have investigated the effects of the reported mutations through RT-PCR followed by sequencing of the RT-PCR resulting product to determine their transcriptional consequences. Table 2 summarizes the 12 *LAMA2 *splice-site mutations with reported transcriptional effects. Of the 12 pathogenic mutations, 10 prompted exon skipping, with three (3/10) resulting in a premature termination codon (PTC). Furthermore, one mutation (1/12) introduced a new acceptor site, leading to a PTC, while another mutation (1/12) caused the inclusion of an intron fragment, also resulting in a PTC. It has been documented that single exon skipping commonly occurs due to mutations at canonical splice sites. However, an alternative splice site may be utilized if the normal splice site is weak or the mutation creates or activates a cryptic splice site. Consequently, the inclusion of an intron fragment can occur if the cryptic splice site is located within an intron, and the exclusion of an exon fragment may happen if the cryptic splice site is present in an exon [48].

**Table 2:** Summary of the reported splice-site mutations and their transcriptional consequences PTC: Premature termination codon

Mutation	Transcriptional Consequences	Reference
c.8244+1G>A	Skipping of exon 58 with PTC	[11]
c.3556-13T>A	New splicing acceptor site resulting in PTC	[17]
c.4058+1G>A	Skipping of exon 27	[17]
c.3924+2T>C	Splicing of exon 26 with in-frame deletion resulting in the loss of 63 amino acids in domain IVa	[23]
5234+1G>A	Skipping of exon 38	[25]
c.5562+5G>C	Insertion of 11 nucleotides of intron 38 within the transcript with PTC and a lower abundance of LAMA2 transcript	[29]
c.2450+5_c.2450+11del	Skipping of exon 17 with PTC at position +4 within exon 18	[33]
c.4524-2A>G	Skipping of exon 31 with PTC in the second domain	[34]
c.397-4_c.478del	Skipping of exon 4	[38]
c.7452-1G>A	Skipping of exon 54	[38]
c.6429+3A>C	Skipping of exons 44 and 45, leading to the loss of the reading frame	[46]
c.4860+2delinsGGCC	Skipping of exon 33 with loss of the reading frame	[47]

The splicing mutations c.397-4_c.478del and c.7452-1G>A were identified in a patient with severe MDC1A. The c.397-4_c.478del mutation resulted in exon-4 skipping, whereas the c.7452-1G>A mutation caused the skipping of exon 54, affecting both the N-terminal domain and G domain of laminin-α2. Together, these mutations accounted for the disease manifestation in the patient who showed a total absence of laminin-α2 expression [38].

The two out-of-frame mutations c.4860+2delinsGGCC and c.6429+3A>C were reported in three cases with LGMD-like phenotype. The c.4860+2delinsGGCC mutation led to the out-of-frame skipping of exon 33. It was found, along with a missense mutation (728T>C), in two patients with a partial expression of laminin-α2 [47]. Based on existing literature, a missense mutation in the N-terminal region of laminin-α2 was reported to be linked with milder clinical presentations, which could partly explain the phenotype of the two cases [5,9,25]. On the other hand, the c.6429+3A>C mutation was found to be responsible for the out-of-frame skipping of exons 44 and 45. It was also identified with an in-frame duplication (exons 4-12) in a case with LGMD-like phenotype [46]. The preservation of the reading frame due to the in-frame duplication (exon 4-12) in this last case could potentially allow for the translation of an abnormal yet partially functional laminin-α2. Along with that, the aberrant transcripts of the alleles bearing two out-of-frame mutations (c.4860+2delinsGGCC and c.6429+3A>C) might have undergone degradation through the nonsense-mediated mRNA decay mechanism [11,19], or the resulting laminin α2 product, if translated, might have been subjected to degradation through the "protein quantity control" mechanism, which could elucidate the LGMD-like phenotype observed in the three cases [11,20].

The mutation c.3924+2T>C led to an in-frame deletion of exon 26, and it was associated with a mild phenotype in two homozygous siblings for this mutation [23]. The mild MDC1A phenotype in both cases could be justified since this mutation permitted the expression of laminin-α2 in the patients, though truncated. Another mutation, c.5562+5G>C, resulted in the inclusion of a portion of intron 38 and a PTC. This mutation and another splice-site mutation, c.9211+6T>C, were responsible for a mild phenotype in one case [29]. The partial expression of laminin-α2 could explain this phenotype. However, the unavailable transcriptional outcome of the c.9211+6T>C mutation remains necessary to explain the case better. It is noteworthy that patients with the same homozygous genotype (c.3924+2T>C and c.3924+2T>C) or compound heterozygous genotype (c.5562+5G>C and 9211+6T>C) exhibited different phenotypes, ranging from severe presentation, where independent walking was not achieved, to milder presentation, in which patients acquired and maintained the ability to walk for years (till last seen) [6,24]. It can be inferred that establishing a clear genotype/phenotype correlation and predicting the prognosis of the disease based on the effect of the mutation is not straightforward in LAMA2-RDs. This complexity and variability arise because other genetic or epigenetic factors may potentially influence the severity of the disease [24,49].

The c.5234+1G>A mutation was reported to cause complete exon 36 skipping in three MDC1A patients: two patients (2/3) with a homozygous genotype (c.5234+1G>A and c.5234+1G>A) and a patient with a compound heterozygous genotype (c.5234+1G>A and c.3085C>T nonsense mutation) who had all a total absence of laminin-α2 [25]. Additionally, six other cases with this mutation and a loss-of-function mutation (nonsense, exonic deletion, and duplication) exhibited a severe MDC1A phenotype [26]. However, two unrelated patients who had the c.5234+1G>A mutation and a missense mutation c.2461A>C presented an LGMD-like phenotype, which is likely attributed to the presence of the missense mutation and a partial laminin-α2 expression [26,27]. This supports the claim that a genotype involving a combination of loss-of-function mutation and missense mutation may lead to a less severe phenotype than genotypes consisting merely of loss-of-function mutations.

The c.2450+5_c.2450+11del and c.4524-2A>G mutations were demonstrated to induce exon skipping, while the c.3556-13T>A mutation led to the introduction of a new splicing acceptor; the three mutations were demonstrated to affect the reading frame resulting thus in a PTC. Conversely, the c.4058+1G>A mutation was merely responsible for exon 27 skipping. The four mutations were identified with another loss-of-function mutation (frameshift or nonsense) in individuals with severe MDC1A [17,33,34]. The transcriptional outcome of the four splice-site mutations correlated with the phenotype of patients who exhibited no expression of laminin-α2. Besides, the resulting phenotype correlated with the entire genotype in these patients since it consisted of loss-of-function mutations.

Our mutation, c.8244+1G>A, introduces a PTC within the highly functional G domain [11]. As previously noted, it has been identified in diverse patients, occurring either in the homozygous state or in combination with other mutations (splicing, frameshift, and nonsense) in the compound heterozygous state. All individuals harboring this mutation manifested a severe MDC1A phenotype, except one patient with mild MDC1A [6,11,18,26,30]. In the cases with severe MDC1A, the severity of the transcriptional impact of this mutation correlated significantly with the resulting phenotype. A study established that this mutation led to a complete absence of laminin-α2, causing a severe MDC1A phenotype [11]. Nevertheless, two patients with a compound heterozygous genotype and a severe MDC1A phenotype demonstrated partial expression of laminin-α2. This variability may be attributed to the impact of the second mutation in each genotype [6]. Nonetheless, additional transcription analyses remain crucial for comprehensively understanding these cases.

In summary, assessing the clinical severity in patients with LAMA2-RDs involves understanding laminin-α2 expression levels and considering the type, location, and mutational mechanism of *LAMA2* mutations. Among the different types of mutations, splice-site mutations play a significant role in LAMA2-RDs, and the clinical severity varies depending on the genotype and transcriptional severity of the disease-causing mutations. The findings regarding the transcriptional consequences of 12 mutations demonstrated that splice-site mutations trigger single exon skipping in most cases but also multiple exon-skipping, insertion of intron fragment, and the activation of a new splicing acceptor site. The pathogenicity of the transcriptional effects of these mutations correlated with the resulting phenotypes, and most of them induced the loss of the reading frame. Thus, a splice-site mutation, which causes the loss of the reading frame when associated with another loss-of-function mutation, primarily results in MDC1A phenotypes. This genotype tends to cause severe MDC1A when combined with a total absence of laminin-α2. In contrast, the same genotype associated with residual laminin-α2 expression leads mainly to a milder MDC1A phenotype. On the other hand, the LGMD-like phenotype is primarily attributed to a splice-site mutation combined with a missense mutation, suggesting that a combination of loss-of-function and missense mutations, with residual laminin-α2 expression, generally results in a less severe phenotype. Nonetheless, exceptions were noted since a combination of loss-of-function mutations resulted in an LGMD-like phenotype, and a splice-site mutation with a missense mutation caused an MDC1A phenotype. A lack of information concerning laminin-α2 expression and the transcriptional consequences of many mutations hinders a comprehensive understanding of these cases. Other than that, it was revealed that the G domain of laminin-α2 is crucial; thus, when aberrant, it results in MDC1A in up to 41% of patients, with no implication in cases of LGMD-like phenotype. Lastly, the observation that the same genotype with the same level of laminin-α2 expression can lead to different phenotypes underscores the potential influence of other genetic or epigenetic factors on the final phenotype and disease prognosis in patients.

## Conclusions

The clinical manifestations of LAMA2-RDs showcase significant diversity, particularly in terms of motor development and disease progression. This diversity poses a considerable challenge in offering precise prognoses, even for individuals sharing identical genotypes. Splice-site mutations play a prominent role among the various mutations implicated in LAMA2-RD. Whether occurring in a homozygous or heterozygous state in association with another loss-of-function mutation, splice-site mutations predominantly lead to severe MDC1A. The severity of the resulting phenotype correlates significantly with the transcriptional consequences of these mutations. Therefore, the transcription analysis of splice-site mutations serves as a crucial tool for comprehending their effects and, consequently, facilitating the establishment of a genotype-phenotype correlation in individuals affected by LAMA2-RDs.

## Appendices

**Table 3:** The documented 57 splice-site mutations and the clinical, paraclinical, and genetic characteristics of the reported 97 individuals ?: Unknown 2nd mutation; A: Absent; CK: Creatine kinase; CNV: Copy number variation; FS: Frameshift; HC: Head control; LGMD: Limb-girdle muscular dystrophy; MDC1A: Congenital muscular dystrophy type 1A; M: month; MS: Missense; NA: Non-available; NS: Non-sense;  P: Partial expression; Pr: Present; R: Running; S: Sitting; Ss: Sitting supported; St: Standing; St. s: Standing supported; W: Walking; Ws: Walking supported; WMC: White matter changes; Y: year

Case	Splice-site mutation	location	Genotype	Second mutation/Location/type	Affected protein domain	Phenotype	Sex	Age at follow up	Age of onset or onset age group	Acquired motor faculties (age)	Loss of motor functions (age)	Level of merosine deficiency	Typical WMC	Other brain abnormalities	Seizures	CK level (UI/L)	Contractures	Scoliosis/ Lordosis	Death	Reference
1	c.112+1G>A	IVS1	Hetero	c.7439+1G>A/ IVS 52/ Splicing	VI+G-like	Severe MDC1A	F	5.5 y	Birth or within 1st week	Bottom shuffle	No	A	Pr	Bilateral occipital micropolygyria	Pr	NA	Pr	Pr	-	[6]
2	c.283+1G>A	IVS2	Hetero	c.391C>T/ Exon3/ NS	VI+VI	Severe MDC1A	M	4.3 y	After 1st week -> before 6th month	Ss	No	A	Pr	Subtle abnormality in cortical folding	A	NA	Pr	A	-	[6]
3	c.283+1G>C	IVS2	Hetero	c.7810C>T/ Exon 56/ NS	LN+ G	Mild MDC1A	M	10,8 y	After 1st week -> before 6th month	HC (>1w to ≤6m), S (10m), W (1.7 y)	No	A	Pr	A	A	343	Pr	A	-	[17]
4	c.283+1G>C	IVS2	Hetero	c.7810C>T/ Exon 56/ NS	LN+ G	Mild MDC1A	F	11,7 y	After 1st week -> before 6th month	HC (>1w to ≤6m), S (10m), W (1.7 y)	No	NA	Pr	A	A	900	Pr	Pr	-	[17]
5	c.640-1G>C	IVS4	Homo	c.640-1G>C/ IVS4/ Splicing	LN+LN	Severe MDC1A	M	8,7 y	Birth	HC (1 y), S (1.5 y)	No	NA	Pr	A	A	1200	Pr	Pr	-	[17]
6	c.640-1G>C	IVS4	Hetero	c.437C>A/ Exon 4/ MS	LN+LN	LGMD	M	3,2 y	1,2 y	HC (3 m), S (7 m), W (1.2 y)	No	NA	Pr	A	A	1344	A	A	-	[17]
7	c.640-1G>C	IVS4	Hetero	c.1580G>A/ Exon 11/ MS	LN+EGF-like	Severe MDC1A	M	6,5 y	Birth	HC (4 m), S (6 m)	No	NA	Pr	A	A	3714	Pr	A	-	[17]
8	c.910-1G>T	IVS6	Hetero	Exon3-4del/ CNV	EGF like+LN	Severe MDC1A	M	6 m	Birth	No HC	No	NA	Pr	A	A	3761	Pr	A	-	[17]
9	c.910-1G>T	IVS6	Hetero	c.3283C>T/ Exon 23/ NS	EGF like+EGF like	Severe MDC1A	M	2 y	Birth	HC (1 y), S (1 y)	No	P	Pr	occipital pachygyria Intellectual delay	A	1246	Pr	A	-	[17]
10	c.910-1G>T	IVS6	Hetero	?	V+?	LGMD	M	NA	2 y	R	No	NA	NA	A	NA	1358	Pr	A	-	[44]
11	c.1207-1G>A	IVS8	Hetero	?	V+?	Mild MDC1A	M	19.5 y	After 6th month ->before 1st year	R	No	P	Pr	A	A	NA	Pr	A	-	[6]
12	c.1207-1G>A	IVS8	Hetero	?	V+?	Mild MDC1A	F	24.5 y	After 6th month ->before 1st year	W	No	P	Pr	A	Pr	NA	Pr	A	-	[6]
13	c.1307-2A>G	IVS9	Hetero	c.6993-6999delTCCTCAG/ Exon 50/ U FS or Splicing	V+G	Severe MDC1A	M	4 m	Birth or within 1st week	NA (examined at 4 m)	NA	A	No (tested in 4 m)	NA	A	NA	NA	A	-	[6]
14	c.2208+2T>C	IVS15	Homo	c.2208+2T>C/ IVS15/ Splicing	IIIb+IIIb	Severe MDC1A	F	3.3 y	Birth or within 1st week	HC	No	A	No (tested in neonatal period)	A	A	NA	Pr	A	-	[6]
15	c.2209-3_2209–2 delCA	IVS15	Hetero	c.624 delC/ FS		Severe MDC1A	F	NA	After 1st week -> before 6th month	No walking	No	NA	Pr	NA	Pr	NA	Pr	NA	-	[32]
16	c.2450+5_c.2450+11del	IVS17	Hetero	c.8007delT/ Exon 57/ FS and PTC inducing	IIIb+ G	Severe MDC1A	F	NA	Birth	St (36 m)	No	A	Pr	Mild mental retardation	NA	1000	Pr	Pr	Died at 10 y due to respiratory complications	[33]
17	c.2450+1G>C	IVS17	Hetero	?	IIIb+?	Severe MDC1A	F	18 y	Birth or within 1st week	Ss	No	A	NA	A	A	NA	Pr	Pr	-	[6]
18	c.2749+2dup	IVS19	Hetero	c.1338_1339del/ Exon 10/ PT causing	IIIb+V	Mild MDC1A	M	3 y	After 6th month ->before 1st year	W (24m)	No	NA	Pr	NA	NA	873	NA	NA	-	[42]
19	c.2749+2dup	IVS19	Hetero	c.7921G>T/ Exon 57/ NS	EGF-like+ G-like	Mild MDC1A	F	1,9 y	After 1st week -> before 6th month	HC (>1w to ≤6m), S (9 m), W (1.5 y)	No	NA	NA	A	A	1978	NA	A	-	[17]
20	c.2749+1G>A	IVS19	Hetero	c.1177T>G/ Exon 8/ MS	IVb+V	LGMD	F	51 y	13 y	R	No	P	Pr	NA	Pr	280	NA	NA	-	[45]
21	c.2749+1G>A	IVS19	Hetero	c.1177T>G/ Exon 8/ MS	IVb+V	LGMD	M	NA	NA	W	No	P	Pr	NA	A	NA	NA	NA	-	[45]
22	c.2749+1G>A	IVS19	Hetero	c.1122delA/ Exon 8/ FS	IVb+V	Severe MDC1A	M	10y	After 6th month ->before 1st year	Walking up to 250 m	No	P	Pr	A	A	4000	NA	A	-	[7]
23	c.3556-1G>A	IVS24	Hetero	Exon9del/ CNV	IV+LN	Severe MDC1A	F	5,6 y	Birth	HC (7 m), S (8 m)	No	NA	Pr	pontine hypoplasia	A	924	Pr	Pr	-	[17]
24	c.3556-13T>A/ FS	IVS23	Hetero	c.4883delC/ Exon 34/ FS	IV+ α1	Severe MDC1A	F	20,8 y	Birth	HC (5 m), S (9 m)	Loss of rolling, (6 y), S (19 y)	NA	Pr	A	A	4479	Pr	Pr	-	[17]
25	c.3556-13T>A/ FS	IVS23	Hetero	c.4883delC/ Exon 34/ FS	IV+ α1	Severe MDC1A	M	9,8 y	Birth	HC (5 m), S (9 m)	No	NA	Pr	A	A	17000	Pr	Pr	Died at 9.8 y	[17]
26	c.3735+2_3735+8delinsAAAGAAGGA	IVS25	Homo	c.3735+2_3735+8delinsAAAGAAGGA/ IVS25/ Splicing	IVa+ IVa	Severe MDC1A	M	6 y	Birth	HC (3 y), S (3 y)	No	A	Pr	A	A	88680	Pr	A	-	[17]
27	c.3924+2T>C	IVS26	Homo	c.3924+2T>C/ IVS26/ Splicing	IVa+ IVa	Mild MDC1A	M	3.5 y	After 1st week -> before 6th month	S(7m); W (26m)	easily stumble	P	Atypical WMC	NA	NA	1187	NA	NA	-	[23]
28	c.3924+2T>C	IVS26	Homo	c.3924+2T>C/ IVS26/ Splicing	IVa+ IVa	Mild MDC1A	F	NA	After 1st week -> before 6th month	W (3y and 8 m)	No	P	Atypical WMC	NA	NA	256	NA	NA	-	[23]
29	c.3924+2T>C	IVS26	Homo	c.3924+2T>C/ IVS26/ Splicing	IVa+ IVa	Mild MDC1A	M	11 y	Birth	W (4y)	No	NA	Pr	NA	NA	603	A	A	-	[24]
30	c.3924+2T>C	IVS26	Homo	c.3924+2T>C/ IVS26/ Splicing	IVa+ IVa	Mild MDC1A	F	9 y	Birth	W (4y)	No	NA	Pr	NA	NA	349	NA	Pr	-	[24]
31	c.3924+2T>C	IVS26	Homo	c.3924+2T>C/ IVS26/ Splicing	IVa+ IVa	Severe MDC1A	M	Birth	Birth	Ss	No	P	Pr	NA	NA	3270	A	A	died at 7 y;	[24]
32	c.3924+2T>C	IVS26	Homo	c.3924+2T>C/ IVS26/ Splicing	IVa+ IVa	Severe MDC1A	M	5 m	After 1st week -> before 6th month	No walking	No	NA	NA	NA	NA	864	Pr	A	-	[24]
33	c.3924+2T>C	IVS26	Homo	c.3924+2T>C/ IVS26/ Splicing	IVa+ IVa	Severe MDC1A	M	3 y, 2 m	Birth	St.s	No	NA	Pr	NA	NA	280	Pr	Pr	Died at 16 y	[24]
34	c.3924+2T>C	IVS26	Homo	c.3924+2T>C/ IVS26/ Splicing	IVa+ IVa	Severe MDC1A	M	13 m	Birth	Ss	No	NA	Pr	NA	NA	1063	Pr	A	-	[24]
35	c.3924+2T>C	IVS26	Homo	c.3924+2T>C/ IVS26/ Splicing	IVa+ IVa	Mild MDC1A	M	7 y	After 1st week -> before 6th month	W (3y)	No	NA	Pr	NA	NA	2506	Pr	NA	-	[24]
36	c.3924+2T>C	IVS26	Homo	c.3924+2T>C/ IVS26/ Splicing	IVa+ IVa	Mild MDC1A	F	4 y	After 1st week -> before 6th month	W (3,5y)	No	P	Pr	NA	NA	1929	Pr	NA	-	[24]
37	c.3924+2T>C	IVS26	Homo	c.3924+2T>C/ IVS26/ Splicing	IVa+ IVa	Mild MDC1A	F	19 m	After 1st week -> before 6th month	W (4y)	No	NA	Pr	NA	NA	49	A	A	-	[24]
38	c.4058+1G>A/	IVS27	Hetero	c.6513_6515del/ Exon 46/ Small del	IV+ G like	Severe MDC1A	M	13,6y	After 1st week -> before 6th month	HC (>1w to ≤6m), S (9 m)	Loss of HC (10 y)	A	Pr	A	A	4436	Pr	Pr	-	[17]
39	c.4058+1G>A/	IVS27	Hetero	c.329G>A/ Exon 3/ NS	IV+ LN	Severe MDC1A	M	13,1 y	Birth	HC (7 m), S (9 m), W (1.5 y)	Loss of W (1.7 y)	A	Pr	pontine hypoplasia	Pr	642	Pr	Pr	Died at 13.1 y following severe pneumonia	[17]
40	c.4311+2T>C	IVS29	Hetero	c.8245-2A>T/ IVS58/ Splicing	EGF-like+ G-like	Severe MDC1A	M	27,3 y	After 1st week -> before 6th month	HC (>1w to ≤6m), S (8 m), W (1.5 y)	Loss of W (8 y)	NA	Pr	Intellectual regression	Pr	1000	Pr	Pr	-	[17]
41	c.4312-1G>A	IVS29	Hetero	del exons 13–14/ CNV Out of frame	IIIa+ IVb	LGMD	NA	NA	Juvenil	W	No	NA	NA	NA	NA	10X	NA	NA	-	[46]
42	c.4524-2A>G	IVS31	Hetero	c.3799_3821del/ Exon 26/ FS	IIIa+ IVa	Severe MDC1A	M	NA	Birth	Walking	Loss of W (6 y)	A	A	diffuse hypomyelination of both hemispheres	Pr	4405	Pr	A	-	[34]
43	c.4523+1G>A	IVS31	Hetero	c.3976C>T/ Exon 27/ NS	IIIa+ IVa	Severe MDC1A	F	4 y	After 1st week -> before 6th month	S	No	A	Pr	NA	NA	2619	Pr	A	-	[35]
44	c.4860+2delinsGGCC	IVS33	Hetero	c.728T>C/ exon 5/ MS	I+II +VI	LGMD	M	NA	23 y	R	No	P	Pr	A	Pr	309	NA	NA	-	[47]
45	c.4860+2delinsGGCC	IVS33	Hetero	c.728T>C exon 5/ MS	I+II +VI	LGMD	F	NA	40 y	R	No	P	Pr	Mild deficit in executive functions	Pr	405	NA	NA	-	[47]
46	4960-2A>G	IVS34	Hetero	c.1545C>A /11/ NS	αI + EGF-like	Severe MDC1A	M	3,1y	Birth	HC (4 m), S (1.5 y)	No	NA	NA	Intellectual delay	A	3385	Pr	A	-	[17]
47	4960-2A>G	IVS34	Hetero	Exon2–3del/Out-of-frame deletion CNV	II+ VI	Severe MDC1A	F	NA	After 1st week -> before 6th month	W	No	NA	Pr	A	A	2427	Pr	A	-	[36]
48	c.5071+1G>A	IVS35	Hetero	Exon2–3del/Out-of-frame deletion CNV	α1+LN	Severe MDC1A	F	12,8y	After 1st week -> before 6th month	HC (>1w to ≤6m), S (10 m), W (4.5 y)	Loss of S (7.5 y), W (6.8 y)	NA	Pr	A	A	2427	Pr	Pr	-	[17]
49	c.5071+1G>A	IVS35	Hetero	Exon2–3del/Out-of-frame deletion CNV	α1+LN	Severe MDC1A	F	13 y	Birth	S (10 m)	NA	NA	Pr	A	A	NA	Pr	Pr	Died at 13 y following severe pneumonia	[17]
50	c.5234+1G>A	IVS36	Hetero	c.3085C>T/ Exon 22/ NS	II+ IIIb	Severe MDC1A	F	4 y	In utero	Play with hands	No	A	Pr	A	A	4460	Pr	Pr	-	[25]
51	c.5234+1G>A	IVS36	Homo	c.5234+1G>A/ IVS36/ Splicing	II+II	Severe MDC1A	M	9 m	Birth	HC	No	A	No (tested in neonatale period)	A	A	4706	Pr	NA	-	[25]
52	c.5234+1G>A	IVS36	Homo	c.5234+1G>A/ IVS36/ Splicing	II+II	Severe MDC1A	F	3 m	In utero	No HC	No	A	Pr	A	A	5530	Pr	NA	-	[25]
53	c.5234+1G>A	IVS36	Hetero	c.2461A>C / exon 18/ MS	II+ LEb	LGMD	M	47 y	After 1st year	R	No	P	Pr	NA	NA	351	NA	Pr	-	[27]
54	c.5234+1G>A	IVS36	Hetero	c.3976C>T/ Exon 27/ NS	II+ IVa	Severe MDC1A	F	NA	NA	No walking	No	NA	NA	A	A	NA	NA	NA	-	[26]
55	c.5234+1G>A	IVS36	Hetero	Exon 56 deletion/ CNV	II+G	Severe MDC1A	M	NA	NA	No walking	No	NA	NA	Intellectual delay	Pr	NA	NA	NA	-	[26]
56	c.5234+1G>A	IVS36	Hetero	c.3085C>T/ Exon 22/ NS	II+ IIIb	Severe MDC1A	F	NA	NA	No walking	No	NA	NA	A	A	NA	NA	NA	-	[26]
57	c.5234+1G>A	IVS36	Hetero	117bpDUP / Exon 38	II+ II	Severe MDC1A	M	NA	NA	No walking	No	NA	NA	A	Pr	NA	NA	NA	-	[26]
58	c.5234+1G>A	IVS36	Hetero	c.391C>T/ Exon 3/ NS	II+VI	Severe MDC1A	M	NA	NA	No walking	No	NA	NA	Intellectual delay	A	NA	NA	NA	-	[26]
59	c.5234+1G>A	IVS36	Hetero	c.2461A>G/ Exon 18/MS	II+IIIb	LGMD	M	NA	NA	W	No	NA	NA	A	A	NA	NA	NA	-	[26]
60	c.5234+1G>A	IVS36	Hetero	c.3976C>T/ Exon 27/ NS	II+ IVa	Severe MDC1A	M	NA	NA	No walking	No	NA	NA	A	A	NA	NA	NA	-	[26]
61	c.5563-1G>A	IVS38	Hetero	c.4048C>T/ Exon 27/ NS	α2 + IV	Severe MDC1A	F	1 y	Birth	HC (7 m), S (8 m)	No	NA	Pr	A	A	2155	A	A	-	[17]
62	c.5562+5G>C	IVS38	Homo	c.5562+5G>C/ IVS38/ Splicing	II+II	Mild MDC1A	F	NA	Birth	W (32 m)	No	P	NA	NA	Pr	3000	NA	NA	-	[28]
63	c.5562+5G>C	IVS38	Hetero	c.9211+6T>C/ IVS64/ Splicing	II+G	Mild MDC1A	F	NA	After 1st week -> before 6th month	W	No	P	Pr	NA	NA	1761	NA	NA	-	[29]
64	c.5562+5G>C	IVS38	Hetero	c.9211+6T>C/ IVS64/ Splicing	II+G	Severe MDC1A	F	2 y	After 1st week -> before 6th month	Ss	No	P	NA	A	A	NA	Pr	A	-	[6]
65	c.5562+5G>C	IVS38	Hetero	c.9211+6T>C/ IVS64/ Splicing	II+G	Mild MDC1A	F	6 y	After 6th month ->before 1st year	W	No	P	Pr	A	A	NA	Pr	A	-	[6]
66	c.5562+5G>C	IVS38	Hetero	c.9211+6T>C/ IVS64/ Splicing	II+G	Mild MDC1A	F	12 y	After 1st week -> before 6th month	W	No	P	Pr	A	A	NA	Pr	A	-	[6]
67	c.5562+5G>C	IVS38	Hetero	c.2049_2050delAG/Exon 14/ FS	II+IVb	Severe MDC1A	M	5 y	After 6th month ->before 1st year	Ws	No	P	Pr	fronto-parietal polymicrogyria	A	NA	Pr	A	-	[6]
68	c.6268+2T>C	IVS43	Hetero	c.5156_5159del/ Exon 36/ FS	αII + αI	Severe MDC1A	F	18,6 y	After 1st week -> before 6th month	S (7 m), W (4 y)	Loss of S (11 y), W (8 y)	A	Pr	A	A	1097	Pr	Pr	-	[17]
69	c.6429+3A>C	IVS45	Hetero	dup: exons 4–12/ CNV In frame	I+?	LGMD	NA	NA	juvenil	W	No	NA	NA	NA	NA	>10X	NA	NA	-	[46]
70	c.6430-2A>G	IVS45	Homo	c.6430-2A>G/ IVS45/ Splicing	I+I	Severe MDC1A	M	NA	Birth	NA	NA	A	NA	NA	NA	1643	Pr	NA	-	[31]
71	c.6867+1G>A=6916+1G>A	IVS48	Hetero	c.2049_2050 del/ Exon 14/ FS	G+VI	Severe MDC1A	M	NA	Birth	Ws	No	A	Pr	A	NA	1585	Pr	Pr	-	[37]
72	c.6993-2A>G	IVS49	Hetero	c.7898G>C/ Exon 56/ Splicing	G-like+G-like	Severe MDC1A	M	2 y	Birth	HC (3 m), S (1 y)	No	NA	Pr	A	A	1200	Pr	A	-	[17]
73	c.6993-2A>C	IVS49	Hetero	c.2049_2050delAG/ Exon 14/ FS	G+ IVb	Severe MDC1A	F	4 y	Birth	Supported trunk control	No	A	Pr	A	A	6987	A	Pr	-	[25]
74	c.7156-2A>G	IVS50	Hetero	c.7147C>T/ Exon 50/ NS	G+G	Severe MDC1A	F	5,3 y	Birth	S (18 m)	No	NA	Pr	A	A	86425	Pr	A	-	[17]
75	c.7452-1G>A	IVS53	Hetero	c.397-4_c.478del/ IVS3_Exon4/ Splicing	G2+ VI	Severe MDC1A	M	5 m	Birth	No walking	No	P	No (tested in 5 m)	NA	NA	1762	Pr	Pr	-	[38]
76	c.7572+1G>T	IVS54	Hetero	c.725G>A/ Exon 5/ MS	G+V	Severe MDC1A	F	13 y	Birth	Sitting (12 m)	No	A	NA	NA	Pr	2391	Pr	Pr	-	[39]
77	c.7750-2A>G	IVS55	Hetero	c.5212G>T/ Exon 36/ NS	G+α1	Severe MDC1A	F	8 m	After 1st week -> before 6th month	No HC	No	NA	NA	A	A	NA	Pr	A	Died at 8 m following severe pneumonia	[17]
78	c.7750-2A>G	IVS55	Hetero	c.5212G>T/ Exon 36/ NS	G+α1	Severe MDC1A	F	1,3 y	After 1st week -> before 6th month	No HC	No	NA	NA	A	A	NA	Pr	A	-	[17]
79	c.7898+1G>A	IVS56	Hetero	c.8931_8933delTCT/ Exon 63/ FS	G+G	Severe MDC1A	F	NA	NA	sitting (2y)	No	P	Pr	NA	NA	2500	Pr	NA	-	[40]
80	c.7898+1G>T	IVS56	Homo	c.7898+1G>T/ IVS56/ Splicing	G+G	Severe MDC1A	M	12 y	After 1st week -> before 6th month	Ss	No	NA	NA	A	A	NA	Pr	Pr	-	[6]
81	c.8244+1G>A	IVS58	Homo	c.8244+1G>A/ IVS58/ Splicing	G+G	Severe MDC1A	F	NA	Birth	Ss	No	A	Pr	A	NA	543	Pr	NA	Died at 10 y	[18]
82	c.8244+1G>A	IVS58	Homo	c.8244+1G>A/ IVS58/ Splicing	G+G	Severe MDC1A	M	2 y	NA	Unable to stand at 2 y	No	A	Pr	A	NA	950	NA	NA	-	[11]
83	c.8244+1G>A	IVS58	Hetero	c.2556delT/ Exon 19/ FS	G+IIIb	Severe MDC1A	M	2 y	Birth or within 1st week	HC	No	P	Pr	NA	A	NA	Pr	A	-	[6]
84	c.8244+1G>A	IVS58	Hetero	c.8988+1G>A/ IVS63/ Splicing	G+G	Severe MDC1A	F	1.3 y	After 1st week -> before 6th month	S	No	P	Pr	NA	A	NA	A	A	-	[6]
85	c.8244+1G>A	IVS58	Hetero	c.2049_2059del/ Exon 14/ FS	G+IVb	Severe MDC1A	F	NA	NA	No Walking	No	NA	NA	A	A	NA	NA	NA	-	[26]
86	c.8244+1G>A	IVS58	Hetero	c.3976C>T / Exon 27/ NS	G+IVa	Mild MDC1A	M	NA	NA	W	No	NA	NA	A	A	NA	NA	NA	-	[26]
87	c.8244+1G>A	IVS58	Hetero	c.6191del / Exon 50/ FS	G+G	Severe MDC1A	F	NA	NA	No walking	No	NA	NA	A	A	NA	NA	NA	-	[26]
88	c.8244+1G>A	IVS58	Hetero	c.1255del/ Exon 9/ FS	G+V	Severe MDC1A	F	NA	NA	No walking	No	NA	NA	A	Pr	NA	NA	NA	-	[26]
89	c.8244+1G>A	IVS58	Homo	c.8244+1G>A/ IVS58/ Splicing	G+G	Severe MDC1A	F	NA	NA	No walking	No	NA	NA	A	Pr	NA	NA	NA	-	[26]
90	c.8244+1G>A	IVS58	Homo	c.8244+1G>A/ IVS58/ Splicing	G+G	Severe MDC1A	M	NA	Birth	St (24m)	No	NA	Pr	NA	NA	18X	NA	NA	-	[30]
91	c.8244+1G>A	IVS58	Homo	c.8244+1G>A/ IVS58/ Splicing	G+G	Severe MDC1A	F	NA	Birth	No walking	No	NA	Pr	NA	NA	NA	NA	NA	-	[30]
92	c.8244+3_8244+6del	IVS58	Hetero	c.7732C>T/ Exon 55/ NS	G+G	Severe MDC1A	F	9,3 y	After 1st week -> before 6th month	HC (>1w to ≤6m), S (8 m), W (3 y)	Loss of W (8.5 y)	NA	Pr	A	A	2025	Pr	Pr	-	[17]
93	c.8244+3_8244+6del	IVS58	Hetero	c.5562+5G>C / Intron 38/ Splicing	G+II	Mild MDC1A	F	NA	After 6th month ->before 1st year	R	No	NA	NA	dysmyelination of the supratentorial white matter	Pr	765	NA	Pr	-	[43]
94	c.8245-2A>G	IVS58	Hetero	c.1122delA/ Exon 8/ FS	G+V	Severe MDC1A	M	NA	After 6th month ->before 1st year	Ss	No	P	Pr	A	A	940	NA	Pr	-	[7]
95	c.8358-3C>G	IVS59	Hetero	c.2959dup/ Exon 21/ FS	G-like+ EGF-like	Severe MDC1A	M	15 y	Birth	HC (10 m), S (14 m)	No	A	Pr	A	Pr	770	Pr	Pr	Died at 15.0 y following epilepsy	[17]
96	c.8703+1G>A	IVS61	Hetero	c.5116C>T/ Exon 36/ NS	G+II	Severe MDC1A	NA	NA	NA	S	No	NA	NA	NA	NA	30000	NA	NA	-	[41]
97	c.8989-12C>G	IVS63	Hetero	c.2451+6 A>G/ IVS17/ Splicing	G+IIIb	Severe MDC1A	F	NA	After 1st week -> before 6th month	W	No	NA	Pr	A	A	NA	NA	NA	-	[32]

## References

[1] Schorling DC, Kirschner J, Bönnemann CG (2017). Congenital muscular dystrophies and myopathies: an overview and update. Neuropediatrics.

[2] Bönnemann CG, Wang CH, Quijano-Roy S (2014). Diagnostic approach to the congenital muscular dystrophies. Neuromuscul Disord.

[3] Muntoni F, Voit T (2004). The congenital muscular dystrophies in 2004: a century of exciting progress. Neuromuscul Disord.

[4] Gawlik KI, Durbeej M (2011). Skeletal muscle laminin and MDC1A: pathogenesis and treatment strategies. Skelet Muscle.

[5] Sarkozy A, Foley AR, Zambon AA, Bönnemann CG, Muntoni F (2020). LAMA2-related dystrophies: clinical phenotypes, disease biomarkers, and clinical trial readiness. Front Mol Neurosci.

[6] Geranmayeh F, Clement E, Feng LH (2010). Genotype-phenotype correlation in a large population of muscular dystrophy patients with LAMA2 mutations. Neuromuscul Disord.

[7] Beytía Mde L, Dekomien G, Hoffjan S, Haug V, Anastasopoulos C, Kirschner J (2014). High creatine kinase levels and white matter changes: clinical and genetic spectrum of congenital muscular dystrophies with laminin alpha-2 deficiency. Mol Cell Probes.

[8] Jones KJ, Morgan G, Johnston H, Tobias V, Ouvrier RA, Wilkinson I, North KN (2001). The expanding phenotype of laminin alpha2 chain (merosin) abnormalities: case series and review. J Med Genet.

[9] Oliveira J, Gruber A, Cardoso M (2018). LAMA2 gene mutation update: toward a more comprehensive picture of the laminin-α2 variome and its related phenotypes. Hum Mutat.

[10] (2024). ClinVar: lama2[gene]. https://www.ncbi.nlm.nih.gov/clinvar/?term=lama2%5Bgene%5D&redir=gene..

[11] Siala O, Louhichi N, Triki C (2007). Severe MDC1A congenital muscular dystrophy due to a splicing mutation in the LAMA2 gene resulting in exon skipping and significant decrease of mRNA level. Genet Test.

[12] LOVD database. (2022 (2024). LOVD database. https://databases.lovd.nl/shared/variants/LAMA2/unique.

[13] (2024). dbSNP: rs749522728. https://www.ncbi.nlm.nih.gov/snp/rs749522728..

[14] Mendell JR, Boué DR, Martin PT (2006). The congenital muscular dystrophies: recent advances and molecular insights. Pediatr Dev Pathol.

[15] Leite CC, Lucato LT, Martin MG (2005). Merosin-deficient congenital muscular dystrophy (CMD): a study of 25 Brazilian patients using MRI. Pediatr Radiol.

[16] Menezes MJ, McClenahan FK, Leiton CV, Aranmolate A, Shan X, Colognato H (2014). The extracellular matrix protein laminin α2 regulates the maturation and function of the blood-brain barrier. J Neurosci.

[17] Tan D, Ge L, Fan Y (2021). Natural history and genetic study of LAMA2-related muscular dystrophy in a large Chinese cohort. Orphanet J Rare Dis.

[18] Louhichi N, Richard P, Triki CH, Meziou M, Ayadi H, Guicheney P, Fakhfakh F (2006). Novel mutations in LAMA2 gene responsible for a severe phenotype of congenital muscular dystrophy in two Tunisian families. Arch Inst Pasteur Tunis.

[19] Nagy E, Maquat LE (1998). A rule for termination-codon position within intron-containing genes: when nonsense affects RNA abundance. Trends Biochem Sci.

[20] Bross P, Corydon TJ, Andresen BS, Jørgensen MM, Bolund L, Gregersen N (1999). Protein misfolding and degradation in genetic diseases. Hum Mutat.

[21] Dirani M, Cuenca VD, Romero VI (2022). COL1A1 novel splice variant in osteogenesis imperfecta and splicing variants review: a case report. Front Surg.

[22] Motavaf M, Soveizi M, Maleki M, Mahdieh N (2017). MYO15A splicing mutations in hearing loss: a review literature and report of a novel mutation. Int J Pediatr Otorhinolaryngol.

[23] Allamand V, Sunada Y, Salih MA (1997). Mild congenital muscular dystrophy in two patients with an internally deleted laminin alpha2-chain. Hum Mol Genet.

[24] Di Blasi C, Bellafiore E, Salih MA (2011). Variable disease severity in Saudi Arabian and Sudanese families with c.3924 + 2 T &gt; C mutation of LAMA2. BMC Res Notes.

[25] Oliveira J, Santos R, Soares-Silva I (2008). LAMA2 gene analysis in a cohort of 26 congenital muscular dystrophy patients. Clin Genet.

[26] Camelo CG, Artilheiro MC, Martins Moreno CA (2023). Brain MRI abnormalities, epilepsy and intellectual disability in LAMA2 related dystrophy - a genotype/phenotype correlation. J Neuromuscul Dis.

[27] Marques J, Duarte ST, Costa S (2014). Atypical phenotype in two patients with LAMA2 mutations. Neuromuscul Disord.

[28] Tezak Z, Prandini P, Boscaro M (2003). Clinical and molecular study in congenital muscular dystrophy with partial laminin alpha 2 (LAMA2) deficiency. Hum Mutat.

[29] Naom I, D'alessandro M, Sewry CA (2000). Mutations in the laminin alpha2-chain gene in two children with early-onset muscular dystrophy. Brain.

[30] El Kadiri Y, Ratbi I, Ouhenach M (2023). Early diagnosis of congenital muscular pathologies using next-generation sequencing: experiences from a tertiary center in Morocco. Egypt J Med Hum Genet.

[31] Fattahi Z, Kalhor Z, Fadaee M (2017). Improved diagnostic yield of neuromuscular disorders applying clinical exome sequencing in patients arising from a consanguineous population. Clin Genet.

[32] Liang WC, Tian X, Yuo CY (2017). Comprehensive target capture/next-generation sequencing as a second-tier diagnostic approach for congenital muscular dystrophy in Taiwan. PLoS One.

[33] Siala O, Louhichi N, Triki C, Morinière M, Fakhfakh F, Baklouti F (2008). LAMA2 mRNA processing alterations generate a complete deficiency of laminin-alpha2 protein and a severe congenital muscular dystrophy. Neuromuscul Disord.

[34] Di Blasi C, van Alfen N, Colleoni F, ter Laak H, Mora M (2007). Severe congenital muscular dystrophy in a LAMA2-mutated case. Pediatr Neurol.

[35] Løkken N, Born AP, Duno M, Vissing J (2015). LAMA2-related myopathy: Frequency among congenital and limb-girdle muscular dystrophies. Muscle Nerve.

[36] Ge L, Liu A, Gao K (2018). Deletion of exon 4 in LAMA2 is the most frequent mutation in Chinese patients with laminin α2-related muscular dystrophy. Sci Rep.

[37] He Y, Jones KJ, Vignier N (2001). Congenital muscular dystrophy with primary partial laminin alpha2 chain deficiency: molecular study. Neurology.

[38] Hashemi-Gorji F, Yassaee VR, Dashti P, Miryounesi M (2018). Novel LAMA2 gene mutations associated with merosin-deficient congenital muscular dystrophy. Iran Biomed J.

[39] Natera-de Benito D, Muchart J, Itzep D (2020). Epilepsy in LAMA2-related muscular dystrophy: an electro-clinico-radiological characterization. Epilepsia.

[40] O'Grady GL, Lek M, Lamande SR (2016). Diagnosis and etiology of congenital muscular dystrophy: we are halfway there. Ann Neurol.

[41] Reddy HM, Cho KA, Lek M (2017). The sensitivity of exome sequencing in identifying pathogenic mutations for LGMD in the United States. J Hum Genet.

[42] Yamamoto-Shimojima K, Ono H, Imaizumi T, Yamamoto T (2020). Novel LAMA2 variants identified in a patient with white matter abnormalities. Hum Genome Var.

[43] Washington C, Stolerman ES, Cooley-Coleman JA, Jones JR, Chen-Deutsch X (2023). RNA analysis of intronic variants in the LAMA2 gene detected by whole genome sequencing confirms a rare dual diagnosis of incontinentia pigmenti with limb-girdle muscular dystrophy. Clin Case Rep.

[44] Yu M, Zheng Y, Jin S (2017). Mutational spectrum of Chinese LGMD patients by targeted next-generation sequencing. PLoS One.

[45] Rajakulendran S, Parton M, Holton JL, Hanna MG (2011). Clinical and pathological heterogeneity in late-onset partial merosin deficiency. Muscle Nerve.

[46] Giugliano T, Savarese M, Garofalo A (2018). Copy number variants account for a tiny fraction of undiagnosed myopathic patients. Genes (Basel).

[47] Gavassini BF, Carboni N, Nielsen JE (2011). Clinical and molecular characterization of limb-girdle muscular dystrophy due to LAMA2 mutations. Muscle Nerve.

[48] Deletang K, Taulan-Cadars M (2022). Splicing mutations in the CFTR gene as therapeutic targets. Gene Ther.

[49] Heydemann A, Doherty KR, McNally EM (2007). Genetic modifiers of muscular dystrophy: implications for therapy. Biochim Biophys Acta.

